# Non-Bleeding Colonic Ulcer as Initial Manifestation of Disseminated Cryptococcosis in a Patient With Human Immunodeficiency Virus

**DOI:** 10.7759/cureus.17298

**Published:** 2021-08-19

**Authors:** Julio C Valencia-Manrique, Adebola Adetiloye, Rasha Alaameri, Jose Flores, Gabriel Ibarra, Yvette Achuo-Egbe, Jennifer Harley, Jagbir Sandhu

**Affiliations:** 1 Medicine, Metropolitan Hospital Center, New York, USA; 2 Gastroenterology and Hepatology, Metropolitan Hospital Center, New York, USA; 3 Pathology, Metropolitan Hospital Center, New York, USA

**Keywords:** cryptococcus, hiv aids, colon ulcer, grubii, disseminated

## Abstract

Cryptococcosis is an invasive mycosis caused by Cryptococcus sp. Its presence is described closely with immunosuppressive states. Once it has reached the body, it has shown a predilection for two sites: the lungs and the central nervous system. Nonetheless, since it has hematogenous dissemination, it can colonize and yield disease at any organ. Hence, a patient will typically present with constitutional symptoms including fever, malaise, and weight loss, associated with cough, shortness of breath, chest pain, or associated headache, drowsiness, and meningeal irritation signs. We illustrate here one of the uncommon non-pulmonary non-cerebral forms of the disease of cryptococcosis, a newly diagnosed HIV/AIDS patient with a non-bleeding colon ulcer, who lacks respiratory or central nervous system (CNS)-related symptoms but endorses non-specific gastrointestinal complaints. The first evidence of the disease was the elevated cryptococcal antigen (CrAg). The direct visualization of the spores in the biopsy confirmed the infection.

## Introduction

Cryptococcosis is an invasive fungal infection that is highly prevalent among immunocompromised individuals. It is known to cause approximately 15% of AIDS-related mortality worldwide [1]. Cryptococcus neoformans is the principal member of the genus. The portal of entry into the organism is primarily the lungs. It colonizes the alveoli and can remain dormant or has a hematogenous spread and potentially invades any organ. Meningitis and meningoencephalitis are the most frequent manifestations of cryptococcosis. Other less affected systems include the gastrointestinal tract, which has been described occasionally in the literature. We present a case of cryptococcosis initially manifested as a non-bleeding sigmoid ulcer in a patient with a previously unknown HIV status.

## Case presentation

**Figure 1:**
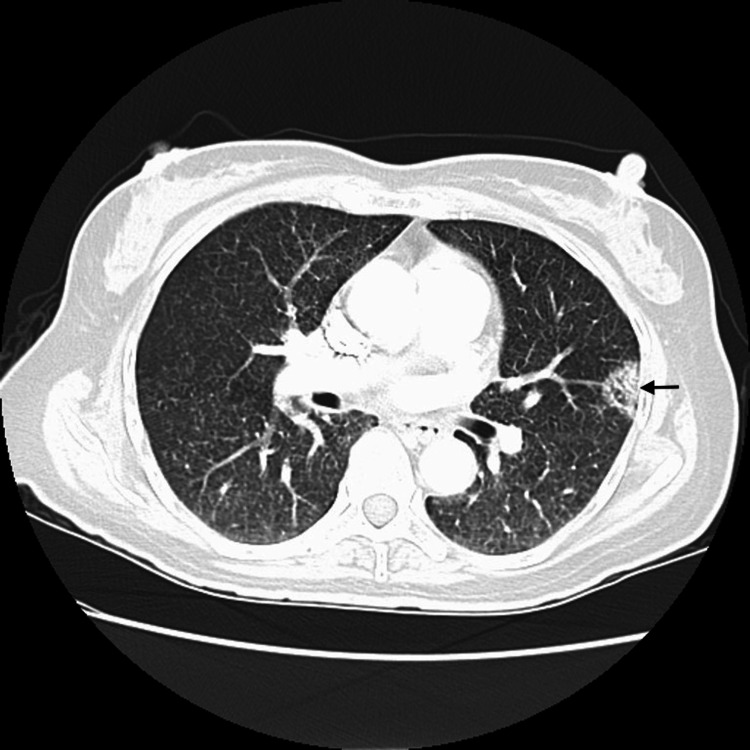
Computerized tomography of the chest without contrast, showing a mass abutting pleura in the apico-posterior segment of the left upper lobe (black arrow). Bilateral tiny reticulo-nodular densities can be seen as well.

A 54-year-old female with a history of hypertension, dyslipidemia, and chronic anemia underwent a colonoscopy and esophagogastroduodenoscopy (EGD) as part of the investigation of concomitant weight loss and retrosternal pain. The studies disclosed a 7mm-ulcer (Figure 1) in the sigmoid colon, whereas the EGD revealed esophageal lesions typical for candidiasis. She was started on oral fluconazole and sent for HIV testing. The patient returned two days later for the results of the test. Upon disclosing the positive results of the HIV test, she suffered a pre-syncopal episode which led her to the emergency room. On further inquiry, she mentioned feeling fatigue, abdominal pain, and mild dysphagia for several months. The physical exam was positive for oral thrush and mild diffuse abdominal tenderness to deep palpation. Meanwhile, the initial blood tests were remarkable for anemia (Hemoglobin 11.5 g/dL) and leukopenia (leukocytes 2440/mcL). EKG was normal. The patient remained under observation for a vasovagal near-syncope and newly diagnosed HIV infection. Additional workup to investigate the sigmoid ulcer was negative for varicella, toxoplasma, histoplasmosis, and T. pallidum. Cytomegalovirus was positive only for the IgG antibody. CD4-count was 19 cells/m^3^ and the viral load 1.3x106 copies/ml. CT abdomen was normal.

The evidence of diffuse bilateral reticulonodular densities in the chest X-ray led to testing for methicillin-resistant staphylococcus aureus/methicillin-sensitive staphylococcus aureus (MRSA/MSSA), respiratory viruses, aspergillosis, legionella, tuberculosis, and fungal infections. All the above-mentioned tests were negative, but cryptococcal antigen (CrAg) was highly positive >1:2560 dilution. P. jirovecii test was not reported due to a processing error. CT chest confirmed the reticulonodular densities and also noted a mass in the left upper lobe (Figure 1). It was considered a possible cryptococcoma.

**Figure 2:**
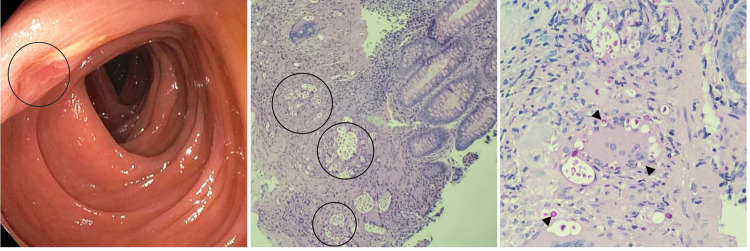
(A) Colonoscopy, a small non-bleeding ulcer observed in the left upper quadrant (black circle). (B & C) Biopsy 40x and 100x, PAS demonstrates the presence of the encapsulated, round-shaped spores (arrowheads) consistent with cryptococcosis, some of them distributed in cluster (black circles). PAS: Periodic acid–Schiff satin

One day after admission, the colon biopsy unveiled chronic inflammatory cells, multinucleated giant cells, and spores of cryptococcus (Figure 2), and the esophageal biopsies confirmed candida esophagitis. In light of the confirmed disseminated cryptococcal infection (elevated CrAg, positive colon biopsy, and possible cryptococcoma), the patient was started on amphotericin 275 mg daily alongside fluconazole 800 mg in addition to prophylaxis for opportunistic infections.

In the following days, the patient started complaining of mild to moderate headaches. It warranted a lumbar puncture to evaluate a possible central nervous system (CNS) compromise. The opening pressure was 28 cmH2O. The fluid was clear, colorless, low in glucose (22mg/dL), and 2% polymorphonuclear cells. The venereal disease research laboratory (VDRL) and the gram stain were negative. Moreover, India ink, meningitis panel-polymerase chain reaction (PCR), and cerebrospinal fluid (CSF) cultures were positive for cryptococcus. These findings confirmed cryptococcal meningitis.

During the hospitalization, she underwent serial imaging of the head to evaluate for elevated intracranial pressure. In addition, she got three more lumbar punctures (LPs), the last showed a normal opening pressure (8cm-H2O), and the CSF culture did not grow any yeast. After six weeks of induction therapy, the patient was transitioned to consolidation therapy and started antiretroviral therapy (ART). The initial symptoms resolved. The patient left the hospital with a plan to follow up in the virology clinic.

## Discussion

Cryptococci are encapsulated yeasts of worldwide distribution. The genus is composed of more than 35 species, among which only two, C. neoformans and C. gattii, have been attributed pathogenic properties. C. neoformans, principally the variety grubii, is responsible for most of the cases of cryptococcosis in immunosuppressed patients, whereas C. gattii has been associated mainly with immunocompetent subjects. C. neoformans proliferates in soil rich in avian droppings. Humans may get infected after being exposed to contaminated vegetation. Inhalation of the basidiospore forms represents the primary route of transmission. Interestingly, the yeast holds a predilection for the central nervous system, which favors brain colonization. Consequently, the presence of respiratory symptoms (accredited to the lungs being the initial site of colonization) or neurologic symptoms (explained by neurotropism) configures the most common manifestation of cryptococcosis.

However once in the body, the yeast can widely disseminate, and be detectable at any organ [2]. Indeed, the first clinical identification of the disease was in a tibia with osteomyelitis. Gastrointestinal cryptococcosis (GIC) has been occasionally reported. Osawa and Singh [3], after a literature review, found 30 cases of GIC reported from 1951 to 2008. Most of the cases of GIC outlines an underlying impaired immune response. HIV/AIDS represents the leading cause of immunosuppression, yet conditions like intestinal lymphangiectasia, immunosuppressive therapy, primary biliary cirrhosis, and malignancies account as alternative sources of weakened immunity. In the settings of HIV/AIDS, gastrointestinal involvement correlates to low CD4 count (<50-100 cell/mm^3^) and typically denotes severe dissemination [3], warranting CNS evaluation regardless of the presence of neurologic symptoms.

In terms of symptomatology, literature refers to GIC as a “mimicker” manifesting as abdominal pain, intestinal mass, mucosal inflammation, mucosal ulcer (as seen in this case), or even isolated iron deficiency anemia. In addition, there is a subgroup of patients which lacks gastrointestinal manifestation, as illustrated by Dromer et al. [4] in a nine-year nationwide epidemiological study in France, and by Washington et al. [5], who determined that 33% of autopsies of known cases of pulmonary and disseminated cryptococcosis were also positive for GIC.

We get to diagnose GIC via endoscopic biopsy, detecting the yeast in the gastrointestinal submucosa. Feces culture is also helpful, but its sensitivity is low. Elevated CrAg is an additional marker that should prompt further testing to evaluate dissemination and CNS compromise [6]. Amphotericin B is the cornerstone of the treatment in the disseminated form, along with flucytosine or fluconazole.

## Conclusions

We conclude that even though GIC is not a usual presentation of cryptococcosis, it should always be considered in patients with any condition hindering the immune system (particularly HIV/AIDS patients) who develop gastrointestinal symptoms and tested negative for more frequent etiologies. GIC should also raise suspicion for dissemination and lead us to evaluate for possible CNS involvement. Elevated CrAg is an additional marker of dissemination and CNS compromise.
